# Biofunctionalized 3-D Carbon Nano-Network Platform for Enhanced Fibroblast Cell Adhesion

**DOI:** 10.1038/srep44250

**Published:** 2017-03-13

**Authors:** A. K. M. Rezaul Haque Chowdhury, Amirhossein Tavangar, Bo Tan, Krishnan Venkatakrishnan

**Affiliations:** 1 Nanocharacterization Laboratory, Department of Aerospace Engineering, Ryerson University, 350 Victoria Street, Toronto, ON, M5B 2K3, Canada; 2 Micro/Nanofabrication Laboratory, Department of Mechanical and Industrial Engineering, Ryerson University, 350 Victoria Street, Toronto, ON, M5B 2K3, Canada; 3 Affiliate Scientist, Keenan Research Centre for Biomedical Science, St. Michael’s Hospital, Toronto, Ontario, M5B 1W8, Canada

## Abstract

Carbon nanomaterials have been investigated for various biomedical applications. In most cases, however, these nanomaterials must be functionalized biologically or chemically due to their biological inertness or possible cytotoxicity. Here, we report the development of a new carbon nanomaterial with a bioactive phase that significantly promotes cell adhesion. We synthesize the bioactive phase by introducing self-assembled nanotopography and altered nano-chemistry to graphite substrates using ultrafast laser. To the best of our knowledge, this is the first time that such a cytophilic bio-carbon is developed in a single step without requiring subsequent biological/chemical treatments. By controlling the nano-network concentration and chemistry, we develop platforms with different degrees of cell cytophilicity. We study quantitatively and qualitatively the cell response to nano-network platforms with NIH-3T3 fibroblasts. The findings from the *in vitro* study indicate that the platforms possess excellent biocompatibility and promote cell adhesion considerably. The study of the cell morphology shows a healthy attachment of cells with a well-spread shape, overextended actin filaments, and morphological symmetry, which is indicative of a high cellular interaction with the nano-network. The developed nanomaterial possesses great biocompatibility and considerably stimulates cell adhesion and subsequent cell proliferation, thus offering a promising path toward engineering various biomedical devices.

Carbon based nanomaterials have received considerable attention by researchers for various biomedical applications such as scaffolds in tissue engineering^1^, substrates for stem cell differentiation^2^, components of implant devices^3^, diagnostic tools and chips^4^, biological imaging^5^, drug delivery carriers^6^, and antibacterial materials^7^. To date, numerous research groups have investigated the response of mammalian cells to carbon nanomaterials in terms of cell viability, adhesion, and proliferation, with most of the recent works have been concentrated on carbon nanotubes (CNTs)^8^ and graphene^9^. Though the carbon nanomaterials have shown some satisfactory results for different cell lines in terms of biocompatibility, their health and safety issues have been a concern, so they have been widely examined for their toxicity. There are still contradictory reports for example for CNTs that show strong cytotoxic activities in some cases and ability to cell growth in some other cases^10^,^11^. Therefore, these nanomaterials usually have to be purified or/and functionalized with chemical/biological molecule for positive cell response owing to their possible cytotoxicity or/and chemical inertness.

In general, the purity of materials influence their cytotoxicity and thus cell responses. As for carbon nanomaterials, the purity particularly differs in materials obtained from carbon nano-compounds^12^. Great number of research works have pointed out that one of the most important factors contributing to the CNT toxicity is impurities that are introduced during synthesis and purification procedures. For instance, CNTs can be produced by several techniques, one of which for large-scale production is chemical vapor deposition (CVD). During CVD, contamination of CNTs by catalyst residues is unavoidable^13^. These impurities lead to cytotoxicity of CNTs. In addition, amorphous carbon is considered an impurity that might result in the toxicity of carbon nanomaterials^12^,^13^,^14^. The impurities thus affect cell responses to the surfaces and eventually influence the cell cultures efficiency. Therefore, effective functionalization processes are usually necessary to purify CNTs while sustaining their original structures^13^. For instance, Mottaghitalab *et al*.^15^ showed that treating single-walled carbon nanotubes (SWCNTs) substrate with the extracellular matrix component fibronectin could reduce these unfavorable characteristics, thus enhancing cellular attachment. In another study, Correa-Duarte *et al*.^16^ showed that a 3-D network of interconnected multi-walled carbon nanotube (MWCNTs) functionalized in an acid solution to generate carboxylic groups was favorable for adhesion and growth of mouse fibroblast cell line L929. Nevertheless, even after the treatment processes, contaminations that increase cytotoxicity may still remain in the resulting product. Besides, the purification method itself may introduce unwanted toxic substances to the nanotubes^12^. In addition, the biofunctionalization stage would add another step to the multistep process of CNT synthesis.

Unlike CNTs, graphene has a much simpler structure and can also be synthesized as a relatively pure layer, which makes it more suitable substrate for the cell growth and differentiation. Indeed, previous studies involving adherent cells have reported the positive impact of graphene on the cell functions^12^. Ryoo *et al*.^17^ employed a glass cover slip coated with a thin film of graphene and/or MWCNTs to adhere NIH-3T3 mouse fibroblasts cells. They showed that graphene improved gene efficiency and focal adhesion and proliferation of fibroblast cells. Aryaei *et al*.^18^ concluded that graphene did not possess any toxic effect on osteoblasts and that the cell adhesion was improved with graphene-coated substrate. In orthopedic applications, graphene polymer composites have also been designed and evaluated^19^,^20^. Depan *et al*. reported that the presence of graphene oxide in chitosan-graphene oxide scaffolds showed enhanced biological responses of osteoblasts, such as cell adhesion, proliferation, growth, and proved its influence in cell scaffold interactions^19^. Shan *et al*. synthesized poly-L-lysine-functionalized graphene a biocompatible and relatively friendly environment due to a large number of free active amino groups^21^. Yet, several researchers have reported that graphene can induce oxidative stress in cultured cells due to the formation of reactive oxygen species on its surface^22^. The induced oxidative stress is believed to contribute to the toxicity of graphene as nanomaterial, so despite positive cell adhesion for particular cell lines, their cytotoxic behavior originated from oxidative stress requires further functionalization^12^,^23^. For instance, several studies have showed that biocompatible graphene could be achieved by chemical functionalization using polymers such as poly(ε-caprolactone)^24^ or poly-L-lysine^21^.

All in all, most carbon nanomaterials/nanostructures synthesized so far require further treatments to increase biocompatibility and to attract cell response. Hence, there is a need for a bioactive carbon with natural functionalization that can trigger cell adhesion and subsequent cell proliferation without the necessity for additional chemical or biological surface treatments.

In this work, we have introduced a novel approach to transform the non-responsive carbon phase of graphite substrate to bio-active carbon phase without the use of post-biological or -chemical treatments. We have utilized a self-assembled bottom-up approach using femtosecond laser ionization of graphite to bio-functionalize graphite substrate through creation of 3-D bio-carbon nano-network platforms. The nano-network is considered 3-D because it is composed of 10–20-μm thick multilayer interwoven nanofiber materials. Laser ionization of graphite, which is performed at ambient condition without the need for vacuum settings, leads to graphite ionization and later formation of nano-network platform, which concurrently alters chemistry and nanotopography of the surfaces. The proposed laser ionization technique is performed in ambient atmosphere and requires no catalyst or background gas (and no chamber). Therefore, no impurity is introduced to the carbon nanomaterial, and thus no post-purification step will be needed. The laser processing parameters can be tuned to create 3-D bio-carbon nano-network platform with desired density and chemistry. The morphology and physicochemical properties of the 3-D bio-carbon nano-network platform have been analyzed using scanning electron microscopy (SEM), field-emission scanning electron microscopy (FE-SEM), energy-dispersive X-ray spectroscopy (EDX), X-ray diffractometer (XRD), X-ray photoelectron spectroscopy (XPS), and micro-Raman spectroscopy. Cell-material interaction and biocompatibility of these 3-D bio-carbon nano-network platform have been investigated with NIH-3T3 fibroblasts (primary cells of connective tissue). Adhesion and morphology of the fibroblast cells have been studied quantitatively and qualitatively using SEM analysis and fluorescence microscopy (FM). Since cell adhesion is a key factor in the subsequent cell activity such as proliferation, protein synthesis, and tissue generation, the significant cell-adhesion-promotor property of the developed nano-network platform suggests considerable outlook for developing numerous carbon-based biomedical devices. Moreover, the simplicity and versatility of the proposed laser technique would make it a promising single-step approach to create biocompatible carbon nano-network platforms through functionalizing the nanotopography and chemistry of graphite substrates.

## Materials and Method

### Carbon nano-network platform fabrication

Isomolded, very fine grain, high strength graphite plate (Graphtek LLC, USA) with 3 mm thickness was cut into 4-cm^2^ squares. These squares were polished with sand papers (3 M Canada, 1000 grit, 2000 grit and 3000 grit) and then ultrasonically cleaned (Cole-Parmer 8890 ultrasonic cleaner) with acetone and ethanol for five min/step and then dried at room temperature. To create a 3-D bio-carbon nano-network platform on the top of these polished surface, these substrates were irradiated with a diode-pumped, Yb-doped femtosecond laser system (Clark-MXR, Inc.; IMPULSE Series ultrashort pulse laser) at laser pulse repetitions of 4, 8 and 26 MHz and atmospheric condition. A fixed stage was used to mount the sample and a computer controlled high precision 2-D translation scanner guided the incident laser beam. An array of lines with a variation of spaces in between was machined to create the 3-D nano-network. The pulse energy transferred to the substrates per unit area -laser fluence (pulse energy/effective focal spot area)-varied at 4.43, 2.22, and 0.68 *J*/*cm*^2^, referred to as high, medium, and low fluences throughout this study, in order to alter the chemistry and nanotopography the developed nano-network. The laser wavelength, pulse width, power of the incident laser beam, the laser beam scanning speed, and irradiation focal spot area were maintained constant at 1030 nm, 214 fs, 15 W, 1 mm/s, and 84.62 × 10^−8^ *cm*^2^, respectively, for all laser pulse. The laser source parameters such as wavelength, pulse width, pulse repetition, and power were controlled and monitored real-time through a computer. The power output was measured before each laser machining to ensure accuracy. The machining parameters, such as laser beam speed or beam dwell time and machining path, were also controlled and monitored through a separate computer, which was connected to the scanner. The functionality and accuracy of the scanner and laser machining parameters were tested before each experiment. All parameters were recorded for each experiment to ensure repeatability and reproducibility.

### Morphological and physicochemical characterization of 3-D network

The morphology of the 3-D bio-carbon nano-network platform was examined by SEM (Hitachi SU-1500) and FE-SEM (Hitachi, SU-8200). Following SEM, EDX was carried out to determine the elemental composition of irradiated graphite. Micro-Raman spectroscopy (Bruker Senterra) (the laser excitation wavelength is at 532 nm), XRD (Rigaku Miniflex 600), and XPS (Thermo Fisher Scientific K-Alpha) were employed to analyze the surface chemistry and material composition of the 3-D bio-carbon nano-network platform.

### Cell Culture

NIH-3T3 mouse fibroblasts cells were grown in a tissue culture flask with Dulbecco’s Modified Eagle Medium: Nutrient Mixture F-12 (DMEM/F12), 10% fetal bovine serum (FBS) and 1% penicillin/streptomycin maintained at 37 °C in 5% CO_2_. Passages between three and six were used for cell studies.

### NIH-3T3 mouse fibroblast cells-material interaction

Prior to seed the cells, the ablated samples were kept under UV light for 20 minutes. The samples then placed inside the Petri dishes containing 3 ml of DMEM/F12 medium and 10% FBS per dish and NIH-3T3 mouse fibroblasts cells were seeded at a density of 10^5^ cells/ml. The Petri dishes were incubated for 24 h and 48 hours. The spent medium was removed, and the samples were fixed with glutaraldehyde after incubation. Subsequently, the sample was washed twice with 1% sodium cacodylate buffer (PH of 7.3) at 4 °C. Then, the cells were dehydrated through a graded ethanol series (from 10% to 100%) for 15 min. Then, the samples were critical point dried and before the SEM examination, samples were sputtered with a gold layer.

### Fluorescent staining of cells

In the first step, paraformaldehyde was used to fix the samples. Then it was followed by incubation in skimmed milk powder to prevent non-specific binding. The samples are then incubated with Alexa Fluor 488 phalloidin (Life Technologies) to stain the actin and cytoskeleton followed by DAPI (4′, 6′-diamidino-2-phenylindole, Life Technologies) to stain the nucleus. An epi-fluorescent Nikon E-400 microscope with FITC and DAPI filter was used, and the data were recorded using a DS-5M-U1 color digital camera (Nikon, Canada).

### Statistics

All experiments were done in triplicate, and the data represented the mean ± standard deviation unless otherwise mentioned. The cell counting was made using SEM micrographs and image processing software.

## Results

### Synthesis of 3-D nanofibrous network on Graphite substrate

**Figure 1:**
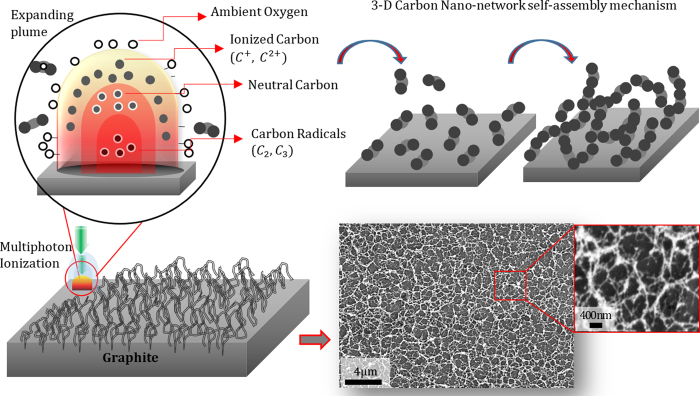
Schematic of the laser ionization of graphite substrate and the synthesis of nano-network platform and its corresponding SEM micrographs.

**Figure 2:**
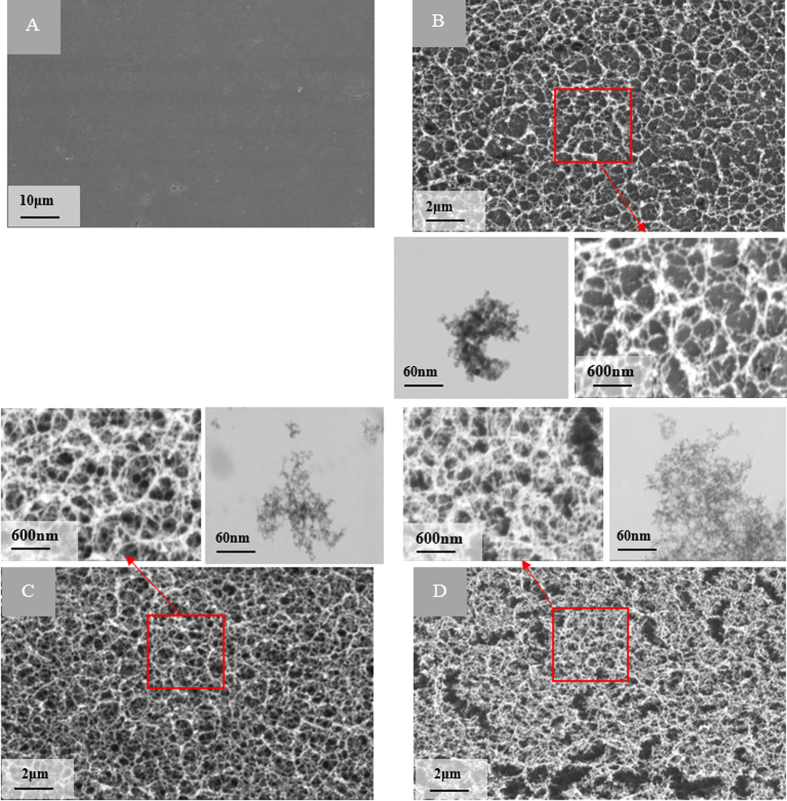
The SEM and corresponding FE-SEM micrographs of (**A**) native graphite substrate and the nano-network platforms created at (**B**) low (**C**) medium, and (**D**) high laser fluence.

The 3-D bio-carbon nano-network platform was synthesized by ultrashort femtosecond laser processing of graphite plates at ambient condition. The schematic of the bottom-up fabrication process is represented in Fig. 1. In general, the formation of nano-network is a function of ionization energy, which directly correlates to laser parameters, such as laser power, ultrashort pulse laser fluence, laser repetition, and scanning speed, background gas (air), and the material properties. In this study the ultrashort pulse laser fluence, which is defined as laser pulse energy per beam area, were tweaked to achieve different nanotopography and chemistry. The change in substrate chemistry is mainly due to the ionization of the graphite by high-energy femtosecond laser pulse. The concentration of created carbon nano-network (*ρ*_*ns*_) is proportionate to the number of ionized carbon nanoparticles (*N*_*np*_) per pulse, which is directly proportional to the ultrashort pulse laser fluence, *f*[*J*/*cm*^2^] = *E*_*p*_[*J*]/*A*_*f*_[*cm*^2^], where *E*_*p*_ is laser pulse energy and *A*_*f*_ is effective laser beam area, and is inversely proportional to laser machining speed^25^, 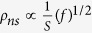. The number of pulses arriving in the same area and thus the transported energy to the substrate can also be controlled with the combination of beam scanning speed and pulse repetition rate. In this study, the ultrashort pulse laser fluence of 4.43, 2.22, and 0.68 *J*/*cm*^2^, high, medium, and low fluence, were employed to create carbon nano-network with three different density and altered chemistry. Figure 2 compares SEM micrographs of a native graphite substrate and the morphology of the nano-network platforms created at low, medium and high fluence with their corresponding FE-SEM micrographs of their constituent nanoparticles.

The SEM micrographs in Fig. 2(A–D) illustrate the evolution of web-like interlinked nano-network on the native graphite surface from low to high ultrashort pulse laser fluence irradiation. In respect to surface morphology, processing with low ultrashort pulse laser fluence resulted in a less dense nanofibrous network with some degree of porosity within it (Fig. 2B). With the increase of ultrashort pulse laser fluence, denser packed nanofibrous structures were synthesized as high energy per pulse led to higher surface ionization, greater nanoparticle generation in the plume, and thus denser aggregation thereof. The FE-SEM micrographs of the fabricated nano-network shown in Fig. 2 also confirmed the interconnected nature of nanoparticles.

**Figure 3:**
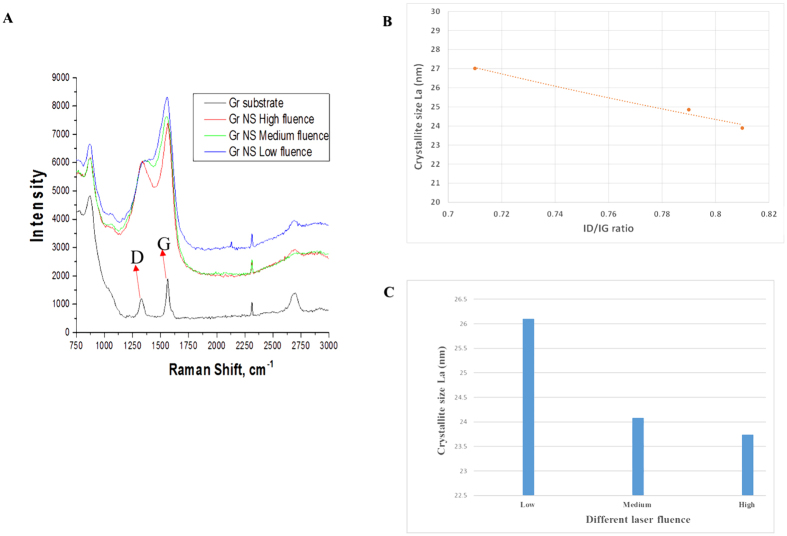
(**A**) A comparison of Micro-Raman spectra of native graphite and the nano-network platform synthesized at different laser fluences, (**B**) the relationships between crystallite size L_a_ and I_D_/I_G_ ratio, and (**C**) the influence of the laser fluences on the crystallite size L_a_. The I_D_/I_G_ ratio is recorded from the Micro-Raman spectra, and it is used to calculate crystallite size L_a_ using Equation 1.

Careful adjustment of laser processing parameters enabled us to achieve, in addition to nanotopography changes, desired chemistry alteration of the nanostructures. Micro-Raman, XPS, XRD, and EDX analyses were conducted to examine the material chemistry of the nano-networks. Micro-Raman spectra of graphite are characterized by two zone center optic phonons that propagate along the crystalline c axis^26^,^27^. The configuration of the sp^2^ sites in the sp^2^ bonded clusters evaluates the dependency of the peaks in the spectrum. E_2_g, the doubly degenerate in-plane optical vibration, is Raman active. For graphite, there are two different E_2_g modes. The difference in energy between the two E_2_g modes has assigned the Raman Bands that is observed in the single crystal of graphite^28^,^29^. G band arises due to the high energy phonon (E_2_g) which corresponds to in-plane, covalent carbon–carbon bond stretching. The low-energy phonon (E_2_g) corresponds to a shearing motion of the weak, inter-planar van der Waals bonds, which shows D band. Figure 3A compares the Raman spectra of a native graphite substrate and the nanofibrous network created with different ultra-short pulse laser fluence. For both native surface and nano network surface, D band peak and G band peak appeared in the range of 1330 cm^−1^ to 1365 cm^−1^ and 1555 cm^−1^ to 1565 cm^−1^, respectively^30^. Two other prominent peaks also appeared at 2314 cm^−1^ and about 2700 cm^−1^, which are known as D’ and G’, and have been previously reported by other researchers^28^. For characterizing nano graphitic structures, the determination of the in-plane crystallite size, which is referred to as *La*, is widely used. An empirical relationship relating *La* and 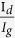 have been used where I_D_ and I_G_ are the intensities of D and G bands. Earlier, Tuinstra and Koenig^29^ performed Raman and XRD studies on different graphitic samples with different in-plane crystallite sizes *La* and concluded that the ratio of the intensities of D and G bands, I_d_/I_g_, was inversely proportional to the crystallite sizes *La*. Based on the experiment of Tuinstra and Koenig [34], later L. G. Cancado *et al*.^31^ developed a general equation of the in-plane crystallite size *La* of crystalline graphite as follows:


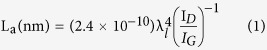


where, λ_l_ is the laser excitation wavelength, I_D_ and I_G_ are the intensities of D and G band. Using I_d_ and I_g_ intensities of D and G band and Equation 1, the in plane crystallite sizes *La* were calculated, as detailed in Table 1. The dependence of the intensities ratio on the crystallite size and effect of the ultrashort pulse laser fluence on the nano-network crystallite formation was graphically presented in Fig. 3B and C.

**Figure 4:**
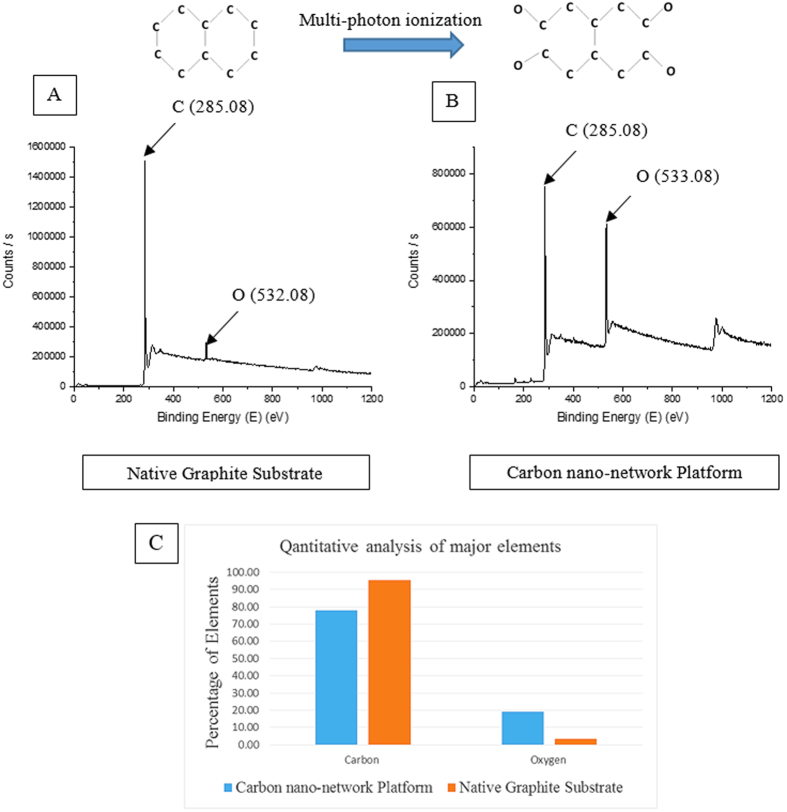
XPS spectra of (**A**) native graphite substrate and (**B**) the nano-network platform; (**C**) XPS quantitative analysis of major elements on both untreated graphite substrate and the nano-network platform created at high fluence.

XPS, FE-SEM-EDX, and XRD analyses were conducted to characterize the surface chemistry and crystal structure of the synthesized 3-D bio-carbon nano-network platform. Figure 4 presents the characteristic XPS spectra of carbon nano-network platform synthesized at high fluence and native graphite substrate, which compare the chemical state of carbon atoms in these two different positions. Two characteristic peaks were observed at 285.08 eV and 533.08 eV for both the carbon nano-network platform and the native graphite substrate. The peak positions 285.08 eV and 533.08 eV in both the carbon nano-network platform and the native graphite substrate can be ascribed to C1s and O1s, respectively^32^,^33^. The quantitative analysis of the major elements as depicted from the graph (Fig. 4C) indicates the increased percentage of oxygen in the synthesized carbon nano-network platform in comparison with native substrate, which is the result of surface ionization in ambient atmosphere. There was a slight, insignificant variance in oxygen content in the created nano-network platforms with the medium and low fluence.

**Figure 5:**
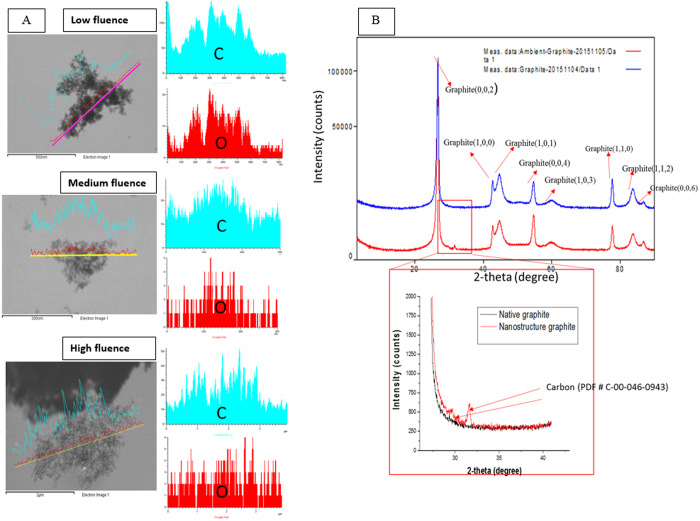
(**A**) FSEM-EDX elemental mapping of the created nano-network platforms at different laser fluences and their corresponding oxygen (*O*) and Carbon (*C*) spectra; (**B**) Comparison of XRD patterns of native graphite substrate and the nano-network platform indicating the presence of standard crystalline nano-graphite pattern as of native graphite except the peak at 2*θ* ≈ 32 which is associated to crystalline carbon (C-00-046-0943).

As shown in the Fig. 5A, the FE-SEM-EDX analysis of the 3-D bio-carbon nano-network platform patterns confirmed they mainly consisted of carbon with a small trace of oxygen. Figure 5B compares the XRD patterns of the native graphite substrate and the 3-D bio-carbon nano-network platform developed on graphite substrates. XRD patterns revealed the presence of standard nano-graphite crystalline orientations as mentioned in Fig. 5B with the exception peak at 2*θ* ≈ 32, which is attributed to crystalline carbon (C-00-046-0943). However, A Marcu *et al*.^34^ reported a similar peak with an almost identical angular position corresponding to the diamond maximal intensity peak (Miller indices - 022).

### Cell interaction with carbon nano-network

**Figure 6:**
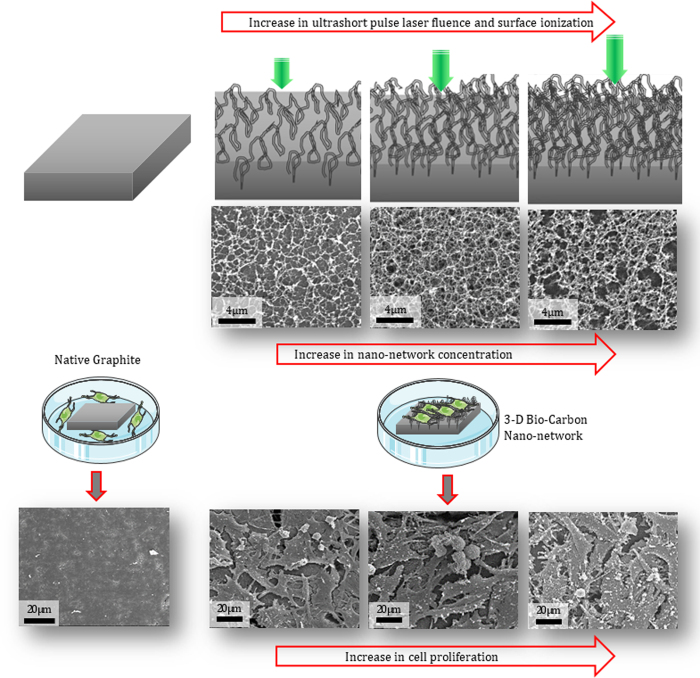
Schematic of native graphite and the nano-network platforms fabricated at different laser fluences and the corresponding SEM micrographs of the substrates before and after fibroblast response after 24 h. (Graphics of the Petri dish and the cells were adapted from Servier Medical Art - creativecommons.org).

Fibroblast cells were used to study the cell adhesion to examine the biofunctionality of the synthesized 3-D bio-carbon nano-network platform. Fibroblasts were seeded on a native graphite substrate and 3-D bio-carbon nano-network platforms with low, medium, and high nano-network concentration, which were created on graphite substrates at different ultrashort pulse laser fluences, as illustrated in Fig. 6.

**Figure 7:**
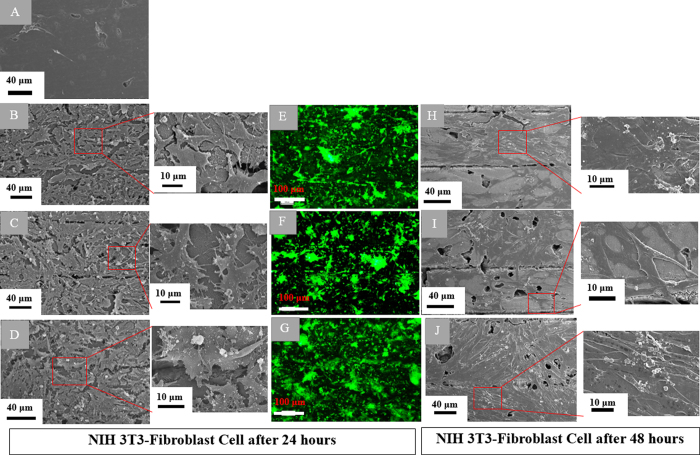
SEM micrographs of fibroblasts adhered on (**A**) native graphite and (**B**–**D**) the nano-network platforms created at low, medium and high fluences, respectively; corresponding fluorescence microscopy images of (**E**–**G**) nano-network platforms created at low, medium and high fluence, respectively after 24 h of culture; and SEM micrographs of fibroblasts cultured on (H-J) low, medium and high fluence mediated nano-network, respectively, after 48 h.

SEM (Fig. 7(A–D) and (H–J)) micrographs of adhered cells were used to assess the cell adhesion and the cell morphological characteristics. Studying the SEM micrographs of the native graphite substrate (Fig. 7 (A)) revealed that no cell was attached to the substrate surface. Whereas, the fibroblasts cultured for 24 h on the 3-D bio-carbon nano-network platform synthesized by low, medium and high ultrashort pulse laser fluence (Fig. 7(B–D)) were attached perfectly. The number of attached cells varied on different substrates with a rising trend from low to high fluence. There was also a morphological symmetry of the cells among those adhered to nano-network. The cells were well adhered to the substrate, flats, proliferative, overlapping each other and covering the entire nano-network platforms for all the cases. The fibroblasts attached to the 3-D bio-carbon nano-network platform were well spread and flattened on the substrate with a morphological symmetry and their filopodia and actin filaments overextended along the nano-network zones, which is suggestive of high cellular interaction with a substrate. Cell area after 48 h of culture for cells adhered on nano-network platforms was significantly greater compared to that after 24 h *in vitro*. Fluorescence images (Fig. 7(E–G)) were coherent with the SEM micrographs. According to the SEM micrographs of cells cultured for 48 h (Fig. 7(H–J)), the density and size of the fibroblasts increased dramatically. The fibroblast attached to 3-D bio-carbon nano-network platform were organized intimately, sometimes overlapping each other. After 48 h of culture, the cells interlinked with each other and formed tissue-like structures. The trend is more prominent on the nano-network created at high laser fluence.

**Figure 8:**
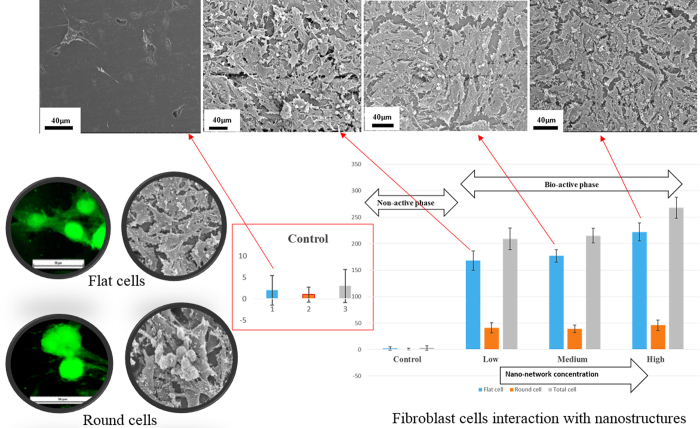
Quantitative analysis of the number of NIH 3T3 fibroblasts adhered on the untreated graphite substrate and the nano-network platforms created at different fluences.

In the next part of the study, we counted the number of cells that adhered on nano-network platforms fabricated at different ultrashort pulse laser fluences in comparison with the native control surface (see Fig. 8). The number of cells attached to the native surface was negligible compared to that of the nano-network areas. Cell morphology could be a good indication of cell response to the substrates, e.g., well-spread polygon shape with extended filopodia is suggestive of healthy cells and increased cellular interaction with a substrate, and round shape is mainly indicative of non-proliferating/apoptotic like cells^35^,^36^. Therefore, we separately screened the number of attached flat cells and round cell to realize the proliferation of healthy cells on nano-network. As observed from the bar graph in Fig. 8, there was an increasing trend in the number of flat cells adhered on the low fluence platform to high fluence one. While, the number of rounded cells showed a steady trend over different platforms.

**Figure 9:**
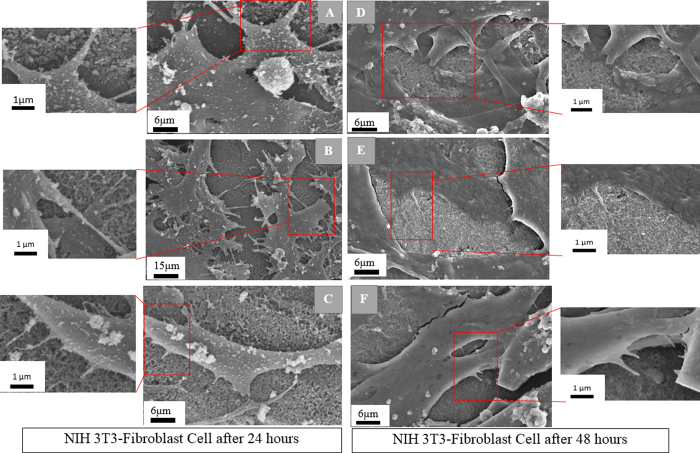
SEM micrographs of the fibroblast cells adhered on low, medium and high fluence mediated nano-network platforms, respectively, after (**A**–**C**) 24 h and (**D**–**F**) 48 h of culture.

SEM micrographs in Fig. 9 depict the response of individual cells with carbon nano-network after 24 and 48 h of growth. The cell adhesion and spreading pattern were almost the same for all the nano-network areas created at different ultrashort pulse laser fluences. The cells were well-adhered with flat shaped, and horizontally extended filopodia over the nano-network, which suggested strong adhesion. The filopodial extensions and anchoring points were shorter for the cells attached on low fluence mediated nano-network after 24 h and not prominent in 48 h (Fig. 9(D)) compared to those adhered on high fluence platform. On the other hand, the long and strong filopodia extensions were observed for medium and high fluence mediated nano-network after both 24 and 48 h.

**Figure 10:**
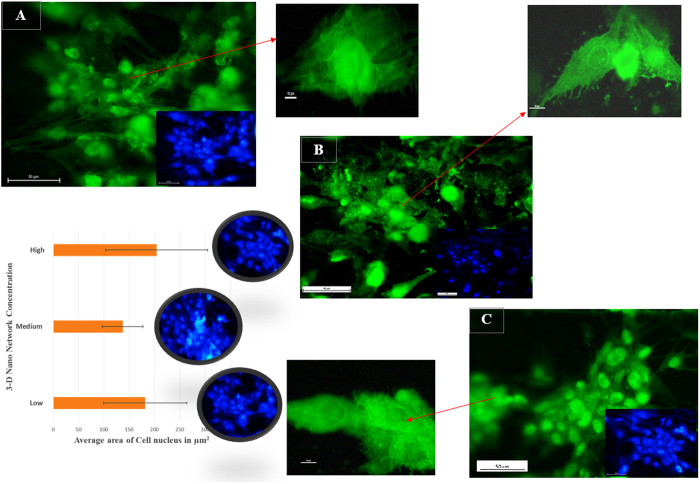
Fluorescence microscopy images and quantitative analysis of the surface area of the nucleus of fibroblasts attached on the platforms created at (**A**) low (**B**) medium, and (**C**) high laser fluence.

To further analyze cell phenotype and nucleus, fluorescence microscopy were employed where actin cytoskeleton and the nucleus of cells were stained in green and blue, respectively. The results were consistent with the SEM observations. The fluorescence data from Fig. 10(A–C) revealed the activity of both cytoskeleton and nucleus of fibroblasts on nano-network synthesized under varying fluence. The fibroblast cells were nicely adhered and grew by spreading over the nanostructured area for all three fluence mediated nano-network. In comparison to low fluence mediated area, the cells were well spread and flat in the other two nano-network (Fig. 10(B,C). Contractile stress fibers, which have been shown to play an important role in cell adhesion and migration^37^, were also observed for cells on all three platforms cultured both for 24 and 48 h, as depicted in Fig. 10. The area of nuclei of the cells for different platforms was also measured and plotted in Fig. 10 for comparing the size of the cells. Since, the cells adhered on the nano-network platforms grew very well and overlapped each other, distinguishing and counting an individual cell was deemed very difficult, and counting the area of cells might lead to inaccurate results. Therefore, the quantitative analysis of the area of the nuclei was performed instead to obtain the most accurate results and correlated those to the cell size, which were in agreement with qualitative results from SEM observation of cells. There is a direct relationship between cell size and nuclear size, and for a given cell type the nuclear/cell volume ratio (karyoplasmic ratio^38^) is said to be constant^38^,^39^,^40^. Though there was a variation in the area of nucleus in the three platforms, the morphological character was the same for all the platforms.

## Discussions

In this study, bio-functionalization of graphite surface were achieved through the synthesis of 3-D bio-carbon nano-network platform on graphite substrates by ultrashort femtosecond laser processing at ambient atmosphere. At the time of interaction between laser pulses with substrate surface on femtosecond time scale, energy deposited into the material faster than needed for the system to react and lead to ion ejection, i.e. ablation from the target. Unlike nanosecond/picosecond laser ablation, femtosecond laser processing has the advantage of having little or no collateral damage due to shock waves and heat conduction produced in the material being processed^41^. The primary mechanism leading to ablation by femtosecond laser pulses were mechanical fragmentation, homogeneous nucleation and vaporization^42^. A high temperature reactive multimodal ablation plasma plume containing neutral carbon (C), carbon radicals (C_2_, C_3_) and carbon ions (C^+^, C^2+^) was generated due to the femtosecond laser irradiation of graphite substrate^43^. The faster-moving carbon ions situate at the top of the expanding plume while heavier radicals and neutral carbon molecules reside at the bottom. The change of fluence from low to high controls the ratios of the plume components and has a significant effect on the growth of nanofibers. With the increment of ultrashort pulse laser fluence, more energy is induced into the plume, which in turn enhances the ratio of carbon ion to other components in the plasma plume. This results in higher level of carbon ionization (C^+^, C^2+^) and increases the number of carbon nano-species in the plasma plume, which will eventually condense and aggregate to form densely packed interwoven nano-network. There was an ascending trend in web-like interlinked 3-D nano-network created in and around to laser irradiated lines with the increase in ultrashort pulse laser fluence due to the indicated reasons, which was evident from the SEM micrographs of the substrates (Fig. 2(B–D)). When plasma plume got outward expansion in presence of ambient atmospheric gas, there was a heat transfer between them and subsequent condensation took place. The oxygen atom that present in the ambient gas and came in contact with the boundary of the plume had a chemical interaction with the carbon ion and there was some trace of C–O molecular bonding presence in the created nano-network, as evidenced by the FE-SEM-EDX analysis in Fig. 5(A).

Micro-Raman spectra of both native graphite substrate as well as the nano-network areas (Fig. 3(A)) showed the graphite specific spectrum with graphite G- Peak and ‘disorder induced’ D peak as referred to elsewhere^44^, although there was a slight deviation of positions of the peaks. The graphite crystallite size *La* gave a focus on the characteristic changes by femtosecond irradiation during recrystallization process, as shown in Fig. 3(B). The nanocrystallite size of the nano-network formed reduces with the increase of laser fluence. The high fluence with more ionization energy resulted in more carbon ions in the plume, which when condensed created widespread nano-network branches of reduced crystallite size, evidenced from the FE-SEM (Fig. 2(D)). Several factors, such as material physical properties, degree of ionization, and the ambient gas characteristics, contribute a remarkable influence on the crystalline structure of the products after laser ablation^41^,^45^. However, since in this study laser ablation is performed in ambient air at atmospheric pressure, the plume expansion, condensation, nucleation, and crystallization are mostly governed by diffusion to the background air rather than adiabatic expansion as in laser ablation in vacuum. Therefore, the characteristics of ambient gas play a much more important role on the crystalline structure of the nanostructures than the degree of ionization/plume temperature, which are slightly affected by laser fluence^41^. This is why the changes in crystal size was only about 10%. Our results from micro-Raman and XPS analyses revealed that there were changes in surface chemistry due to the presence of C–O molecular bond along with the C–C bonds in the created nano-network. Surface of carbon nano-network platform had higher amount of oxygen in comparison with native substrate, which was the result of surface ionization at very high temperature in ambient atmosphere. As our findings showed, the nano-network carbon consisted of crystalline carbon with larger crystalline size compared to native graphite. XRD analysis also confirmed the dominance of C–C bonds in the resulted 3-D bio-carbon nano-network platform with the presence of standard nano-graphite crystalline orientations which was in line with standard graphite diffraction pattern.

Our studies on fibroblast cell response to the synthesized 3-D bio-carbon nano-network platform showed that the interwoven nano-network were effectively affirmative in adhering the cells compared to the native graphite surface where the same cells were seeded for 24 h. The native graphite surface has failed to generate enough favorable cues for initial attachment of the fibroblast cells. According to recent studies, the adhesion of cells to a substrate can be divided into two basic steps: initial attachment and adhesion^46^. The change in surface nanotopography resulted from 3-D nano-network platform and surface chemistry because of the presence of C–O bond in the nano-network provided stimulation for cell adhesion. The resulted nano-network surface is believed to improve adsorption of different extracellular matrix proteins such as fibronectin, vitronectin and collagen, which ultimately helps cell adhesion^47^. These molecules might be adsorbed from the serum of the culture medium in such an appropriate amount with favorable conformation that cell adhesion receptors, e.g., integrins, are able to access specific amino acid sequences for affirmative cell adhesion^48^,^49^,^50^. Again, in the biological interactions of nanomaterials, surface area plays a key role^51^,^52^. The bio-carbon nano-network platform synthesized at high fluence posse greater degree of interconnected nanofibers, which results in a higher surface area with reduced crystallite size compare to that of low fluence-mediated nano-network. This might give a significant amount of surface exposure for protein adsorption and eventually more sites for cells adhesion^53^. The extracellular matrix (ECM)-like morphology of the nano-network, which provides the tissue-like micro-environment for the cells, promote subsequent cell adhesion and proliferation.

In order to evaluate the biocompatibility of bio-carbon nano-network platform, a quantitative analysis of the number of NIH 3T3 fibroblast cell adhered on nano-network platforms at different fluence conditions (low, medium and high) was performed (Fig. 8). The cells adhered on the nano-network areas were flat and well adhered. The number of rounded cells was very less compare to the flat cells. Normal morphological characteristics of flat cells with well-formed cytoskeleton and filopodia indicated the biocompatibility of bio-carbon nano-network platform. Also, the fact that all the cell that adhered on the nano-network platform synthesized with low, medium and high fluence formed stress fibers, as evident from Fig. 10, implied that the resulted nano-network provided favorable cues for initiating stress fibers. On the other hand, the rounded cells that were counted mostly resided on the top of the other flat cells and in agglomerated form. These cells might not have found a proper site or contact to start proliferation. Therefore, the findings suggested the positive proliferative nature of the developed nano-network platform and confirmed that it promoted robust fibroblast growth. It is expected that the proposed approach to bio-functionalize carbon surface advance the development of new biomedical devices for diverse biomedical applications, such as tissue engineering, orthopedic and dental implants, and drug delivery devices.

## Conclusion

In this work, we reported the developing a new bio-carbon nano-network platform with significant proliferative property on fibroblast in a single step without the use of post-biological or -chemical treatments. We applied a bottom-up approach based on ultrafast laser ionization to bio-functionalize graphite substrate via the creation of self-assembled 3-D carbon nano-networks. Ultrafast laser ionization of graphite resulted in graphite ionization and generation of 3-D bio-carbon nano-network platform with modified nano-chemistry and nanotopography. Different degrees of cell proliferations were achieved by controlling the transferred ultrashort pulse laser energy to the surface, which directly altered both the concentration and nano-chemistry of the nano-network. The *in vitro* biocompatibility of the fabricated nano-network was studied quantitatively and qualitatively with fibroblasts. The findings from SEM and FM analyses confirmed strong attachment and growth of healthy fibroblast cells on the graphitic carbon nano-network compared to the untreated counterpart. The cell morphology and adhesion patterns were nearly the same for all the nano-network areas fabricated at different ultrashort pulse laser fluences. The number of cells adhered on the high fluence-fabricated platform in 24 h was 91-fold more compared to untreated graphite, which indicated the high proliferative nature of the developed nano-network. Interlinked tissue-like structures were observed after 48 h of culture, where no single cell was clearly identifiable. The results of this study suggest that the developed nano-network platform not only is biocompatible but also encourages fibroblast proliferation vigorously. The simplicity of the proposed technique to rapidly transform biologically-inactive graphite to biocompatible cell-adhesive substrates will make it a unique approach for developing bio-functionalized carbon-based platforms for the use in biosensing, bioimaging, cancer therapy, and tissue engineering.

## Additional Information

**How to cite this article:** Chowdhury, A. K. M. R. H. *et al*. Biofunctionalized 3-D Carbon Nano-Network Platform for Enhanced Fibroblast Cell Adhesion. *Sci. Rep.* **7**, 44250; doi: 10.1038/srep44250 (2017).

**Publisher's note:** Springer Nature remains neutral with regard to jurisdictional claims in published maps and institutional affiliations.

## Figures and Tables

**Table 1:** Crystallite size (La) calculation using Equation 1 and the I_d_ and I_g_ intensities from Micro-Raman spectra of nano-network platforms developed at different fluences.

	I_d_	I_g_	I_d/_I_g_	λ	L_a_
Low	6339.16	8913.18	0.71	532	27.01
Medium	6549.79	8342.73	0.79	532	24.85
High	5975.28	7412.35	0.81	532	23.89
